# Effects of Neurological Disorders on Bone Health

**DOI:** 10.3389/fpsyg.2020.612366

**Published:** 2020-11-30

**Authors:** Ryan R. Kelly, Sara J. Sidles, Amanda C. LaRue

**Affiliations:** ^1^Research Services, Ralph H. Johnson VA Medical Center, Charleston, SC, United States; ^2^Department of Pathology and Laboratory Medicine, Medical University of South Carolina, Charleston, SC, United States

**Keywords:** Neurology, disease, osteoporosis, bone, mental health, depression, PTSD

## Abstract

Neurological diseases, particularly in the context of aging, have serious impacts on quality of life and can negatively affect bone health. The brain-bone axis is critically important for skeletal metabolism, sensory innervation, and endocrine cross-talk between these organs. This review discusses current evidence for the cellular and molecular mechanisms by which various neurological disease categories, including autoimmune, developmental, dementia-related, movement, neuromuscular, stroke, trauma, and psychological, impart changes in bone homeostasis and mass, as well as fracture risk. Likewise, how bone may affect neurological function is discussed. Gaining a better understanding of brain-bone interactions, particularly in patients with underlying neurological disorders, may lead to development of novel therapies and discovery of shared risk factors, as well as highlight the need for broad, whole-health clinical approaches toward treatment.

## Introduction

The skeleton is necessary for facilitating movement, providing a framework for muscles and soft tissues, protecting vital organs, storage of minerals and fat, harboring the bone marrow, and regulating blood cell formation. Maintaining bone health is, therefore, important for overall health and well-being. In recent years, we have begun to better dissect the relationship between the brain and skeleton and how they regulate one another. One way to better appreciate this relationship is to determine how it becomes altered with disease. Osteoporosis is the most common form of metabolic bone disease and is characterized by low bone mass (≤ 2.5 standard deviations below peak bone mass) and micro-architectural bone deterioration that can lead to debilitating fractures. In the United States, osteoporosis accounts for over 1.5 million fractures annually. By 2050, the costs associated with osteoporotic fractures are expected to exceed $130 billion (Bartl and Bartl, 2019). In regard to brain disease, more than one million U.S. adults are diagnosed annually with a chronic brain disease or disorder at a healthcare cost of > $800 billion (Gooch et al., 2017). In addition, many of these disorders are associated with increased age, a shared risk factor with osteoporosis. Osteoporosis is more prevalent in people with neurological conditions, such as multiple sclerosis (MS) and Parkinson’s disease (PD), while sudden neurological events, such as stroke and spinal cord injury (SCI), can cause rapid loss in bone mineral density (BMD). Further, lack of physical activity in degenerative neurological conditions can lead to mineral loss and osteoporosis, particularly in aging populations and/or those with comorbidities (e.g., obesity, diabetes, smoking). A better understanding of the mechanisms that cause bone loss and how neurological conditions uniquely impact bone health is of clinical importance and will guide treatment options, encourage lifestyle change, and aid in development of novel osteoporosis therapies. Likewise, gaining a better understanding of how bones regulate the brain may provide novel insights into how neurological disorders develop. This review will provide an overview of literature related to neurological disorders and their effects on bone health with a goal to promote recognition of the mechanisms by which changes in the brain can impart changes in the bones. We also discuss how certain treatments for neurological disorders can negatively affect bone health. This will encourage a broader view of disease management toward improved patient health and outcomes.

## Bone-Brain Interface

As an adaptation to stress and to maintain calcium homeostasis, the skeleton undergoes constant remodeling. In adult humans, the skeleton replaces itself almost entirely every 10 years [Office of the Surgeon General (US), 2004]. During this remodeling process, the new bone laid down by osteoblasts must replace the bone resorbed by osteoclasts in a fine-tuned manner. However, with age, net bone loss occurs as a result of increased osteoclastic activity and/or decreased osteoblastic activity at a rate of approximately 1% per year after 30 years of age. The bone remodeling process is regulated by endocrine, paracrine, mechanical, and, as the subject of more recent focus, neuronal factors.

Investigation into the contribution of neuronal signaling in regulating bone remodeling and homeostasis began with a focus on leptin, an adipose-derived hormone involved in energy regulation and metabolism. Mice deficient for leptin (*ob/ob* mice) were found to have a high vertebral trabecular bone mass phenotype (Ducy et al., 2000). This phenotype was, surprisingly, rescued by infusion of small amounts of leptin in the third hypothalamic ventricle (Thomas et al., 1999; Cornish et al., 2002; Takeda et al., 2002). It became evident that the pathways connecting brain and bone play critical roles in energy regulation and bone metabolism. Since this landmark finding, a rapid expansion in studies attempting to understanding this brain-bone interface occurred, with both the sympathetic (SNS) and parasympathetic nervous systems (PNS) having now been shown to regulate bone through various pathways, including leptin, serotonin, adiponectin, circadian genes, neuropeptide Y, muscarinic receptors, nicotinic receptors, beta-adrenergic receptors, and sensory nerve innervation of bone (Marenzana and Chenu, 2008; Dimitri and Rosen, 2017; Elefteriou, 2018). The importance of autonomic tone in regulating bone mass is supported by the observed benefit of beta-blockers on BMD and fracture risk (Schlienger et al., 2004; Bonnet et al., 2007; Graham et al., 2008; Reid, 2008).

Psychological stress can also impart changes in bone. We have previously discussed the relationship between osteoporosis and psychological stress, which is partially regulated through activation of the hypothalamic-pituitary-adrenal (HPA) axis, glucocorticoid signaling, and blunted response of growth factors that contribute to bone mass, such as insulin-like growth factor-1 (IGF-1) (Kelly et al., 2019). Conversely, bone has been found to regulate the brain, with osteocalcin (OCN), an osteogenic hormone, being essential for activation of the acute stress response via inhibition of the PNS (Berger et al., 2019). Thus, the brain-bone axis is critically important for skeletal metabolism, sensory innervation, and endocrine cross-talk between these organs.

Low BMD and cognitive impairment/reduced executive function often occur together, with osteoporosis and related fracture being associated with progression of cognitive impairment, likely, in part, due to increased fall risk (Liu-Ambrose et al., 2007; Cauley et al., 2016). In community-dwelling adults aged > 50 years without history of stroke or dementia, low BMD correlated with cognitive impairment, even after adjusting for confounding factors (Kang et al., 2018). Cognitive impulsivity is significantly related to BMD in elderly women, while verbal working memory has been associated with BMD and may alter fracture risk (Martino et al., 2019; Catalano et al., 2020b). The incidence of physical frailty and dementia follow similar patterns in the aging population, providing further evidence that frailty and cognitive function may be mechanistically and pathologically linked (Halil et al., 2015). Possible mechanisms for the association between BMD and cognitive impairment have been proposed. Mineral concentrations in osteoporosis may facilitate formation of senile plaques and neurofibrillary tangles (NFT) in Alzheimer’s disease (AD) (Lopez et al., 2008; Loskutova et al., 2009; Zhou et al., 2011). Estrogen, involved in bone homeostasis, may also affect cognitive function through inhibition of neuronal apoptosis, promotion of synaptic plasticity, reduction of oxidative stress, and improvement in cerebral blood flow by increasing high-density lipoprotein cholesterol levels (Funk et al., 1991; Brownbill and Ilich, 2004; Zhou et al., 2011; Kang et al., 2018). Further, estrogen replacement may lead to benefits in cognitive function (Luine, 2014). Age-related alterations in inflammatory processes, including increased levels of circulating IL-6 and diminished T regulatory cell activity, may dually contribute to bone loss (via local activation of osteoclasts) and cognitive decline (via increasing levels of neuronal α2-macroglobulin, a protease inhibitor implicated in AD plaques) (Ershler, 1993; Licastro et al., 2000; Lui et al., 2003). Lastly, parathyroid hormone (PTH) may represent a clinically important link between cognitive dysfunction and bone health, as a regulator of calcium and phosphate metabolism that can cross the blood-brain barrier (Lourida et al., 2015; Murthy et al., 2018). Hyperparathyroidism has been associated with physical frailty, bone mass loss via osteoclast activation, as well as poor cognitive function, potentially through calcium overload, disruption of neuronal signaling, and hippocampal atrophy (Numann et al., 1984; Roman et al., 2005; Marcocci et al., 2012; Murthy et al., 2018). It is clear that the bone and brain play key roles in regulating each other. Understanding how bone phenotypes are altered with neurological disorders may provide further mechanistic insights.

## Neurological Disorders

To date, there are more than 600 classified neurological disorders with distinct etiology, neuro-pathophysiology, and symptomology. Herein, we discuss major categories of neurological disorders, including autoimmune, dementia-related, infection-related, movement-related, neural developmental, neuromuscular, psychological, trauma-related, vascular, and other neuronal diseases. Although the etiology of many neurological disorders is highly complex and not fully understood, genetic, epigenetic, and external (e.g., trauma, infection, environment) factors have been implicated in neurological disease initiation and progression. Considerable clinical evidence across many neurological disease categories indicates that changes in neurophysiology lead to changes in bone physiology, resulting in decreased BMD and bone mineral content (BMC), altered bone microarchitecture, and decreased bone strength, ultimately leading to the development of osteopenia/osteoporosis and increased fracture risk. Recent clinical and preclinical studies have begun to shed light on some of the shared hormonal, immune, and molecular/cellular pathways that are impacted in neurological disorders and may mediate secondary effects in bone (Table 1). These include sustained activation of peripheral and central nervous systems (CNS), inflammatory pathways, changes in glutamate signaling, dysregulation of the SNS and PNS, and HPA axis dysregulation. In addition to shared pathophysiology, lifestyle changes secondary to neurological disorders, including modifications in diet and overall physical activity, can contribute to deleterious bone effects. Further, drug treatments for many neurological disorders (e.g., selective serotonin reuptake inhibitors (SSRIs), antipsychotics, anti-epilepsy drugs) have independent and overlapping roles in mediating bone loss. Since osteopenia/osteoporosis and resulting fracture have debilitating effects on patient quality-of-life, it is imperative that we understand how neurological diseases lead to secondary effects in bone to move toward more informed, comprehensive clinical treatment.

**TABLE 1:** Neurological disorders, impacts on bone, and potential mechanisms.

Neurological Disorder	Impact on Bone		Potential Molecular/Cellular Drivers
	
	↓ BMD	↑ Fracture Risk	
**Acute Spinal Cord Injury**	✓	✓	CGRP, OPG, PTH, RANKL, Vasoregulation, VIP, Vitamin D, Wnt
**Alzheimer’s Disease**	✓	✓	Aβ, ApoE, Calcium, Collagen, ERK, GPR158/OCN, IκB-α, OPG, OPN, PNS signaling, RANKL, Serotoninergic input, SOST, TREM2, Wnt/β-catenin
**Amyotrophic Lateral Sclerosis**	✓	✓	Akt, β-catenin, Calcium, Erk1/2, Neurotoxic metals, RANKL, SOST, Vitamin D
**Ataxia**	✓	✓	Frataxin, Immune dysregulation, Vitamin D
**Autism Spectrum Disorder**	✓	✓	IGF-1, OCN, Prolactin, Vitamin D
**Cerebral palsy**	✓	✓	Calcium, IGF-1, PTH, SOST
**Chronic Fatigue**	✓	✓	IGF-1, Macrophages, MALP-2
**Epilepsy**	✓	✓	ALP, BALP, Calcium, Collagen, OCN, PTH Sodium, Vitamin D
**Major Depressive Disorder**	✓	✓	ACTH, Calcium, IL-1β, IL-2, IL-6, Leptin, OPG, OPN, PTH, RANKL, SNS signaling, TNF-α, Vitamin D
**Multiple Sclerosis**	✓	✓	IL-1, IL-6, IL-11, IL-17A, OPN, PNS signaling, PTH, SNS signaling, TNF-α, Vitamin D
**Myasthenia Gravis**	✓	✓	Acetylcholine, Vitamin D
**Neuromuscular Dystrophy**	✓	✓	Calcium, Dystrophin, IL-6, IL-11, Inhibin-βA, OCN, OSX, RANKL, SMN1/2, TGFβ2, Vitamin D
**Parkinson’s Disease**	✓	✓	Homocysteine, Lewy body deposition, PTH, Vitamin D
**Post-Traumatic Stress Disorder**	✓	✓	β-adrenergic stimulation, Catecholamines, Glucocorticoid signaling, IGF-1, IL-1β, IL-6, NF-kB, RANK/RANKL, TNF-α
**Sepsis/SAE**	✓	?	Collagen, Macrophages, Monocyte precursors, Myelopoiesis
**Sleep Disorders**	✓	?	Cortisol, CRP, CTX, Hypoxic signaling, IL-6, P1NP, TNF-α, Vitamin D
**Stroke**	✓	✓	Estrogen, Neuroinflammation, OPG, Vitamin D
**Substance Abuse Disorder**	✓	✓	ALP, BALP, BGP, LDL, Leptin, Vitamin D
**Traumatic Brain Injury**	✓	✓	Calcium, Collagen, IGF-1, NF-κB, OCN, PTH, Vitamin D
**Vertigo**	✓	✓	Calcium, Estrogen, SNS signaling, Vitamin D

*Neurological disorders are associated with negative impacts on bone. These effects may be mediated by primary pathophysiology, lifestyle changes as a result of neurological disease, and first- and second-line therapies. Clinically supported evidence of impacts on BMD and/or fracture risk are marked by “✓.” Unknown or understudied effects are noted by “?.” Molecular/cellular targets and signaling pathways implicated in mediating effects on bone are listed for each disorder.*

### Autoimmune Disorders

#### Multiple Sclerosis

Multiple sclerosis is the most common, non-traumatic disease impacting young adults and can be divided into two stages: an inflammatory phase leading to relapsing-remitting disease and a secondary phase leading to prolonged neurodegeneration and non-relapsing, progressive disease (Dobson and Giovannoni, 2019). Early disease is characterized by neural inflammation, leading to the production of demyelinating plaques in the CNS that results in episodes of vision loss, fatigue, and tingling/numbness. As MS progresses, irreversible axon damage occurs and patients experience pain, muscle spasms, vision loss, and trouble walking. While the etiology of MS has not been fully elucidated, it likely involves multiple factors in addition to genetic predeterminants, including smoking, vitamin D, obesity, gut microbiota, and Epstein-Barr virus (EBV) infection (Dobson et al., 2012; Michel, 2018).

Clinical evidence suggests that MS is associated with detrimental bone effects, including osteopenia/osteoporosis and increased fracture risk (Moen et al., 2011; Sioka et al., 2011; Coskun Benlidayi et al., 2015; Huang et al., 2015a; Bisson et al., 2019). Meta-analyses show that MS patients have reduced BMD in lumbar spine, femur neck, and hip regions compared to healthy controls (Huang et al., 2015a). In a large clinical study, the prevalence of osteoporosis was found to be significantly higher in MS patients (16–26%) compared to healthy controls (6–15%) after adjusting for potential confounding factors, including age, sex, previous fracture history, and comorbidities (Bisson et al., 2019). Assessment of bone microarchitecture in MS patients, reported as a trabecular bone score (TBS), revealed no significant changes compared to healthy controls, suggesting that bone density, but not microarchitecture, is impacted in MS (Olsson et al., 2018).

Potential risk factors for reduced BMD in MS patients include vitamin D insufficiency, disease duration (> 7 years), total steroid dose (> 15 g), disease severity, decreased ambulation, altered parasympathetic signaling, and inflammation (Coskun Benlidayi et al., 2015; Huang et al., 2015b; Murphy et al., 2016). In female MS patients, BMD of lumbar spine and femoral neck was found to be 1-2 SDs lower than in age-matched, healthy controls, with lowest BMD values observed in patients with most severe disease (Nieves et al., 1994). BMD was directly correlated to levels of circulating vitamin D and inversely correlated with levels of parathyroid hormone (PTH), pointing to vitamin D deficiency and hyperparathyroidism as potential contributors to bone loss. In another study of both female (pre- and post-menopausal) and male MS patients, significant bone loss was observed in the femoral neck region, with female patients also presenting with bone loss in the spine. Compared to 2% of healthy controls, 22% of these patients had experienced a non-trauma-related fracture since the age of 35, highlighting a significant clinical outcome (Cosman et al., 1998). Both ambulatory status and steroid treatment > 5 months were shown to be predictors of bone loss, and patients with low levels of vitamin D exhibited greater bone loss overall. Importantly, bone loss in the spine was only observed in patients with low vitamin D levels and was insignificant in patients with normal vitamin D levels. In relatively young, ambulatory patients experiencing acute MS relapses, 51% were found to have low BMD and 62% were found to be vitamin D-deficient, providing further support for a potential link between bone health and vitamin D status (Murphy et al., 2016).

Disease severity and duration, as well as decreased ambulation, have been associated with bone loss and increased risk of fracture in MS patients (Tyblova et al., 2015; Olsson et al., 2018). MS patients with moderate disease, defined by a score of < 4.5–6.5 > on the Expanded Disability Status Scale (EDSS), had lower BMC and BMD in total and regional scans compared to patients with mild disease (score < 1.0–4.0 >) (Pilutti and Motl, 2019). In studies of pre-menopausal women with MS, a significant inverse relationship was observed between EDSS score, disease duration, and BMD, also pointing to disease severity as a primary predictor of bone loss (Zorzon et al., 2005; Terzi et al., 2010). Importantly, lifetime dosage of glucocorticoids was not associated with bone loss in this population, but only with EDSS score (Zorzon et al., 2005). In male patients, decreased mobility and lower EDSS scores were associated with decreased BMD in the femur and muscle wasting in the lower extremities (Zikán et al., 2012). Chronic use of glucocorticoids was not associated with bone loss in this population.

Inflammation has been implicated as a driver in MS-related bone loss. Several inflammatory factors implicated in the pathogenesis of MS, including IL-1, IL-17A, TNF-α, IL-6, and IL-11, have been shown to play a role in osteoporosis (Rifas, 1999; Kasper and Shoemaker, 2010). Levels of proinflammatory osteopontin (OPN) are increased in MS patients compared to healthy controls, with levels directly correlating with femur neck bone loss (Altıntaş et al., 2009). In relapsing-remitting MS patients, increased levels of circulating OPN were shown to correlate with levels of circulating IgG and markers of bone turnover, pointing to OPN as a potential driver of MS-related bone loss (Vogt et al., 2010). Increased levels of OPN were also observed in the cerebrospinal fluid (CSF) of MS patients and correlate with cognitive impairment, suggesting OPN may be a key player in the dysregulated bone-brain axis in MS and may serve as a biomarker of disease progression (Wen et al., 2012). In addition to pathological inflammatory processes, alterations in SNS and PNS signaling have been observed in MS patients and, in some cases, correlate with disease severity and progression (Flachenecker et al., 1999, 2001; Elefteriou, 2008). In a cross-sectional study, patients with active relapsing-remitting MS exhibited impaired sympathetic function and decreased levels of circulating catecholamines compared to healthy control and clinically stable patients (Bartl and Bartl, 2019). Longitudinal follow-up of a subset of these patients revealed a progressive decline in parasympathetic, but not sympathetic, function, suggesting that autonomic tone is differentially impacted as the disease progresses. Future studies in MS patients will be required to delineate the direct and indirect impacts of autonomic dysfunction on bone, as well as how bone innervation and/or signaling may change with disease progression. Together, these studies indicate that pathological mechanisms mediating primary MS symptoms and impaired mobility play major roles in mediating bone loss in MS patients, while the effects of glucocorticoids on bone in MS may be minimal and potentially offset by the positive impact of restored mobility.

### Dementia

#### Alzheimer’s Disease

Alzheimer’s disease is a progressive neurodegenerative disorder and the primary cause of dementia. AD currently affects over 5.8 million Americans age 65 and older, and this is anticipated to grow to 13.8 million by mid-century (Alzheimer’s Association, 2020). AD is characterized by progressive loss of cognitive function and pathologically by extracellular senile plaques enriched in amyloid-β peptide (Aβ) and intracellular NFTs formed by hyperphosphorylated tau protein (Mandelkow and Mandelkow, 2012; Selkoe et al., 2012). For over 20 years, skeletal fragility has been recognized as a comorbidity in AD (Birge et al., 1994; Melton et al., 1994; Johansson and Skoog, 1996; Weller, 2004; Looker et al., 2012). Low BMD and osteoporosis occur at twice the rate in AD patients as neurotypical adults, and this is independent of age, sex, body mass index, physical activity, and disease stage (Melton et al., 1994; Weller, 2004; Loskutova et al., 2009; Zhou et al., 2011, 2014; Zhao et al., 2012). Bone loss has been shown to occur in pre-clinical AD, often preceding diagnosis, thus it may have predictive value in estimating AD risk and likelihood of progression to full AD in patients with mild cognitive impairment (MCI) (Tan et al., 2005; Zhou et al., 2011, 2014; Chang et al., 2014; Sohrabi et al., 2015). Low BMD values have been suggested to predict a faster and more severe rate of cognitive decline (Zhou et al., 2014). Thus, while bone loss cannot be used as an independent risk factor for AD, as it frequently occurs in the non-dementia population, it may add predictive value to models used to assess dementia risk.

Regarding molecular pathways that intersect brain and bone in AD, the major genetic risk factor for AD is the apolipoprotein E (ApoE) 4 allele (Raber et al., 2004). ApoE also plays a critical role in maintaining bone mass by promoting osteogenesis and inhibiting osteoclastogenesis (Noguchi et al., 2018). Transgenic mouse models of AD, including the htau, amyloid precursor protein (APP)/presenilin1 mutant, and Swedish mutation APP strains exhibit low BMD (Cui et al., 2011; Yang et al., 2011; Xia et al., 2013; Peng et al., 2014; Dengler-Crish et al., 2016). Studies using the htau mouse model showed evidence of low BMD that preceded the onset of widespread tauopathy and memory deficits (Dengler-Crish et al., 2016, 2018). Tauopathy in the dorsal raphe nucleus (DRN) localized within serotonergic neurons and was associated with a 70% reduction in the overall number of serotonergic neurons in htau DNR, suggesting a link between serotoninergic input, bone loss, and AD. In AD mouse models that overexpress Aβ (e.g., *APPswe* and *APP/PS1ΔE9*), low BMD and osteoporosis were reported (Yang et al., 2011; Zhou et al., 2014). APP and its cleavage fragment Aβ are expressed in both neural and non-neural tissues, including osteoblasts and osteoclasts, and studies show that Aβ can directly impair osteoblast proliferation and promote osteoclast activity (Cui et al., 2011; Xia et al., 2013). Mechanistically, Aβ was shown to enhance RANKL-induced osteoclast activation through IκB-α degradation, ERK phosphorylation, and calcium oscillation signaling pathways (Li et al., 2014).

Clinical evidence suggests that dysfunctional autonomic signaling contributes to AD-related bone loss. AD patients commonly exhibit increased sympathetic tone and reduced parasympathetic flow, marked by reduced cholinergic innervation in the aging population (Aharon-Peretz et al., 1992; de Vilhena Toledo and Junqueira, 2010; Schliebs and Arendt, 2011). Further, AD patients treated with AChE inhibitors exhibit reduced risk of hip fracture and improved bone healing, suggesting that impaired parasympathetic signaling impacts bone homeostasis in AD patients and may be targeted to improve bone health (Weller, 2004; Tamimi et al., 2012; Eimar et al., 2013).

A potential common contributor to both AD and bone loss is the wingless-type murine-mammary-tumor virus integration site (Wnt) signaling pathway. In brain, Wnt signaling plays a role in neuronal survival and formation of synaptic connections and has been reported to play a neuroprotective role in AD (Oliva et al., 2013). In bone, Wnt signaling through the canonical (i.e., Wnt/β-catenin) pathway promotes osteoblast differentiation and increased bone mass (Krishnan, 2006). Mechanistically, this pathway influences renewal of stem cells, stimulation of pre-osteoblast replication, induction of osteoblastogenesis, and inhibition of osteoblast and osteocyte apoptosis (Krishnan, 2006). Loss-of-function mutations in the Wnt signaling pathway results in skeletal fragility and decreased bone mass (Shah et al., 2015). Preclinical studies in the htau mouse demonstrated Wnt signaling deficiencies in both the brain and bones of mice with low BMD (Dengler-Crish et al., 2018). Triggering receptor expressed on myeloid cells-2 (TREM2) is one potential activator of the canonical Wnt/β-catenin pathway that may tie together bone and brain effects. TREM2 is expressed on microglia, where it is neuroprotective, and on osteoclasts, where it controls the rate of osteoclastogenesis (Otero et al., 2012; Jay et al., 2015; Bemiller et al., 2017). Homozygous loss-of-function mutations in TREM2 are associated with an autosomal recessive form of early-onset dementia presenting with bone cysts and consequent fractures called Nasu–Hakola disease (Paloneva et al., 2000). Meta-analysis has shown heterozygous rare variants in TREM2 are associated with a significant increase in the risk of AD (Guerreiro et al., 2013). Together, these studies suggest a common potential target for addressing bone loss in AD.

While the studies above highlight the potential mechanisms regulating brain’s influence on bone, an understanding of bone’s effects on brain is emerging. Bone is considered an endocrine organ that influences other organs through the secretion of proteins, such as OCN, sclerostin (SOST), and OPN. Blood biomarkers associated with osteoporosis, including C-terminal collagen fragments, OPG, and OCN, are increased in AD (Emanuele et al., 2004; Luckhaus et al., 2009). OCN is a bone-derived hormone that can regulate brain development and function (Greenhill, 2013). Circulating OCN inversely correlates with age, and cognitive function of aged mice can be improved with injection of plasma from young mice (Villeda et al., 2014). Most recently, Karsenty’s group has identified the G protein-coupled receptor 158 (GPR 158) as a receptor for OCN in the brain (Khrimian et al., 2017; Obri et al., 2018).

Sclerostin is an osteocyte-specific secreted glycoprotein, encoded by the SOST gene that binds to low-density lipoprotein-receptor-related protein-5 or -6 (LRP5/6) to regulate Wnt signaling. Through this binding, SOST prevents Wnt ligand binding to LRP5/6 and its co-receptor, Frizzled, leading to decreased bone formation and increased bone resorption (Shah et al., 2015). While these data suggest SOST may impact Wnt signaling, which, in turn, affects the brain and AD pathophysiology (Inestrosa et al., 2002; Inestrosa and Varela-Nallar, 2014), further research needs to be conducted, as it is unclear if circulating SOST can cross the blood-brain barrier (BBB). One potential mechanism by which SOST could influences brain physiology may be through vascular regulation. SOST has recently been shown to influence vascular pathophysiology, a known risk factor for dementia, with high levels of SOST having been linked to cardiovascular mortality (Justin et al., 2013; Novo-Rodríguez et al., 2013; Catalano et al., 2020a). However, whether or not SOST plays an associative or causative role in vascular pathophysiology, and how this may influence development of dementia, remains unknown.

Osteopontin is thought to enhance bone resorption by anchoring osteoclasts to bone matrix, and high serum levels of OPN correlate with low BMD in post-menopausal women (Reinholt et al., 1990; Cho et al., 2013; Fodor et al., 2013). OPN also acts as a cytokine with upregulated production in response to inflammation and injury, including neuronal damage (Denhardt et al., 2001; Wang and Denhardt, 2008). OPN levels increase in patients with MCI progressing to AD, suggesting OPN could be a marker of neuroinflammation and early clinical stages of AD (Simonsen et al., 2007; Sun et al., 2013). Thus, it is possible that, early on, increased OPN expression may be neuroprotective in AD. This is supported by studies demonstrating a more marked increase in OPN levels in AD subjects in early stages of disease (Comi et al., 2010).

Shared risk factors for AD and bone loss include aging, systemic inflammation, depression, genetics, sex, and physical inactivity. However, the relationship between bone loss and AD is complex and cannot be solely attributed to aging, osteoporosis, or dementia. Rather, data suggest there are common pathophysiological mechanisms contributing to both diseases. Further adding to this complexity is the reciprocal crosstalk that occurs between brain and bone. Thus, bidirectional signaling between brain and bone tissue should be considered in the context of AD and its treatments.

### Infection

#### Septicemia/Sepsis

Septicemia, the entry of bacteria into the bloodstream, leads to rapid immune activation and can result in a systemic reaction (sepsis), which can lead to death (Taeb et al., 2017). While the pathophysiology of sepsis is complex, it involves an impaired immune response in which an initial, rapid increase in inflammation is followed by sustained dysregulation of immune activation/suppression, impacting multiple organ systems and resulting in long-term morbidity (Uhle et al., 2016; Taeb et al., 2017; Cecconi et al., 2018). Increased activation of the peripheral immune system can also lead to CNS inflammation, owing to a disrupted BBB, which can result in sepsis-associated encephalopathy (SAE) (Meneses et al., 2019). As SAE impacts roughly 70% of septic patients and is a leading cause of brain dysfunction, it is critical to understand the mechanisms by which sepsis-induced dysregulation of the neuroimmune-endocrine response may impact organ systems like bone (Lamar, 2011).

Clinical evidence suggests sepsis negatively impacts bone health. In a retrospective study of patients initially treated for sepsis with absolute increase > 2 in Sequential Organ Failure Assessment score, significant bone loss was observed in the thoracic, lumbar, and sacral spine regions (Hongo et al., 2019). A second retrospective study showed that sepsis patients had an increased risk for developing osteoporosis compared to non-sepsis patients (Lee et al., 2020). In addition to bone loss, sepsis has been linked to heterotopic ossification, the abnormal formation of lamellar bone in connective tissue. In a retrospective study of patients hospitalized for burn injuries, sepsis following burn injury was associated with the development of heterotopic ossification ∼37 days after admittance, suggesting sepsis temporally modulates bone physiology (Orchard et al., 2015). Together, these studies indicate that the mechanisms by which sepsis impacts bone are complex and likely dependent on many factors, including type, duration, and location of initial infection.

Studies in preclinical models have shown sepsis rapidly reduces bone strength, impacts cellular differentiation in bone marrow (BM), and induces prolonged changes in peripheral macrophage populations. In a rat model of cecal ligation-puncture, trabecular bone strength was significantly reduced beginning 24 h following sepsis induction and was associated with decreased collagen and mineral elastic modulus at 24- and 96-h post-sepsis induction, respectively. These results suggest sepsis rapidly impacts biomechanical properties of bone and may lead to lasting changes in bone microarchitecture (Puthucheary et al., 2017). In addition to altered biomechanics, data indicate sepsis impacts differentiation of BM myeloid cells. In a combined rodent model of burn injury and sepsis, a shift in myeloid differentiation toward monocytopoiesis 72 h following thermal injury was reported, indicating sepsis can lead to rapid changes in differentiation of HSCs in BM (Santangelo et al., 2001). Alterations in the BM compartment were also observed in a rodent model of cecal ligation-puncture, in which epigenetic modifications in BM-derived monocyte precursors were found to impact the function and wound-healing capabilities of circulating macrophages (Davis et al., 2019). These studies point to modulation of monocyte differentiation and macrophage function as additional mechanisms by which sepsis may induce long-lasting effects in bone, possibly through dysregulated osteoclast differentiation and/or alterations in osteal macrophage function.

### Movement Disorders

#### Parkinson’s Disease

Parkinson’s disease (PD) is a progressive neurodegenerative basal ganglia syndrome characterized by bradykinesia and rigidity, resulting in limited daily activity and increased fall risk (Latt et al., 2009; Tassorelli et al., 2017). A number of studies have examined impacts of PD on bone, with PD being associated with decreased BMD and increased fracture risk (Vaserman, 2005; Wood and Walker, 2005; Fink et al., 2008; Gnädinger et al., 2011; Raglione et al., 2011; van den Bos et al., 2013; Gao et al., 2015; Sleeman et al., 2016). Meta-analysis indicates PD patients are at a higher risk for osteoporosis and have lower hip, lumbar spine, and femoral neck BMD compared to healthy controls (Zhao et al., 2013). Women with PD have 7.3% lower total hip BMD and an increased risk of hip fracture (Schneider et al., 2008). Vitamin D concentrations and weight loss are reduced in early PD patients and associated with bone loss (van den Bos et al., 2013; Ozturk et al., 2020). No difference was found in BMD between male PD subjects with short disease duration (0 to 5 years) compared to those with longer disease duration (5 to 10 years), suggesting PD progression may not correlate directly to decreasing BMD and that early detection is key to addressing PD-induced bone loss (Daniel et al., 2012). Thus, PD patients should be closely monitored for vitamin D levels and weight, as well as receiving routine dual-energy X-ray absorptiometry (DEXA) scans and fracture risk assessment (FRAX) (Henderson et al., 2019).

Evidence for molecular mechanisms of PD related to bone loss and increased fracture risk is scant. Lewy body deposition in areas of the brain that regulate bone growth and strength may play a role (Litvan et al., 2007; Daniel et al., 2012). Lifestyle changes associated with PD may result in vitamin D deficiency, which can impact bone loss via compensatory hyperparathyroidism (Invernizzi et al., 2009; van den Bos et al., 2013; De Pablo-Fernández et al., 2017). PD also alters levels of bone metabolism markers (Sato et al., 2004; Bezza et al., 2008). Elevated homocysteine levels from levodopa treatment, the central drug treatment for PD, may also impact bone, as homocysteine can induce osteoclast differentiation and osteoblast apoptosis (Koh et al., 2006; Lee et al., 2010). A significant proportion of PD patients suffer from depression, and concomitant use of antidepressants with levodopa results in a 3- to 5-fold increase in risk of hip and femur fracture (Lieberman, 2006; Arbouw et al., 2011). In general, several factors may be involved in development of bone loss associated with PD, including limited mobility/activity, malnutrition, low body mass index, decreased muscle strength, medication use, and vitamin D deficiency (Invernizzi et al., 2009; Malochet-Guinamand et al., 2015). Larger and more powerful studies are needed to determine effects of PD on osteoporosis risk and to stratify this risk by various confounding factors.

#### Ataxia

Ataxia, a degenerative CNS disease, results in impaired balance and coordination. It is usually caused by damage to the cerebellum but can be caused by damage to the spinal cord or other nerves. There have been few studies on subtypes of ataxia and their effects on bone. In Friedreich ataxia (FDRA), the most common inherited ataxic disorder in the Caucasian population caused by a GAA triplet expansion in the first intron of the frataxin gene on chromosome 9q13, scoliosis and foot deformities are frequent (Labelle et al., 1986; Delatycki et al., 2005; Milbrandt et al., 2008). A strong negative correlation between ataxia severity, GAA repeat length, and BMD was reported in the femoral neck of FDRA patients (Eigentler et al., 2014). The low observed BMD may be due to disease-related falls, mobility restrictions, and/or wheelchair-dependency. Additionally, vitamin D levels were low in the patient cohort, but it is unclear as to why. Suboptimal bone growth and mineralization in FRDA patients during childhood and adolescence may also impact adult BMD and growth. Farias et al. assessed BMD in patients with spinocerebellar ataxia type 3, also known as Machado-Joseph disease (MJD), which is a progressive ataxia resulting in movement restriction caused by an abnormal cytosine-adenine-guanine (CAG) expansion on chromosome 14q32.1. Ten patients out of thirty showed low BMD in at least one of the sites studied, while five patients had at least one lumbar fracture and seven patients reported more than ten falls per month (Farias et al., 2019). This study also found a correlation between CAG expansion and low femoral neck score, providing further evidence that gene alterations may be related to lower BMD. Simonsen et al. found that 75.3% of patients with hereditary ataxia had osteopenia or osteoporosis (Simonsen et al., 2016). These studies suggest the need for routine BMD measurements in ataxia patients to initiate prophylactic osteoporosis treatments. Further studies are needed to determine molecular and genetic mechanisms as opposed to lifestyle changes (e.g., reduced exercise/mobility, increased falls) that may be causing bone loss. In addition, patients with ataxia telangiectasia show immune dysregulation and premature aging, both of which can affect bone loss (Ambrose and Gatti, 2013). Examining immune markers and comparing bones from young ataxia patients to bones from the elderly may provide new pathways for study.

### Neural Development Disease

#### Autism Spectrum Disorder

Autism spectrum disorder (ASD) is a neurodevelopmental disorder with heterogeneous origin and symptomology, including atypical autism, autism, and Asperger Syndrome, that disproportionately impacts males (3:1) (Kim et al., 2011; Sinha et al., 2015; Bhandari et al., 2020). Main symptoms include lack of social interaction, abnormal emotional/sensory processing, and repetitive, restricted behaviors, while secondary symptoms can include irritability, anxiety, aggression, and comorbid disorders. The etiology of ASD is complex and likely involves variations in genes regulating synaptogenesis and signaling pathways, as well as epigenetic and environmental factors, that ultimately lead to neural plasticity dysfunction and the precipitation of social, emotional, and sensory processing symptoms.

Poor nutrition, decreased physical activity, vitamin D deficiency, and use of antipsychotic therapies (APTs) have been associated with poor bone health in ASD. In a cross-sectional study of adolescent boys aged 8–17 years, ASD patients exhibited lower BMD in the lumbar spine, femoral neck, total hip, and whole-body regions compared to age-matched controls (Neumeyer et al., 2013). In addition to lower BMD scores, adolescent boys with ASD had lower consumption levels of protein, calcium, and phosphorus, and were less physically active than typically developing adolescents. In another study of prepubertal boys, patients with ASD were shown to have lower BMD at both the hip and femoral neck regions compared to healthy control patients, as well as lower levels of serum vitamin D and decreased physical activity, pointing to vitamin D deficiency and decreased overall activity as potential risk factors for ASD-related bone loss in the adolescent ASD population (Neumeyer et al., 2013).

Autism spectrum disorder also impacts bone microarchitecture and fracture risk. In a cross-sectional study of adolescent boys, ASD patients exhibited lower trabecular thickness, compressive stiffness, and failure load at the ultradistal radius, as well as a 61% reduction in cortical area compared to typically developing controls, with similar effects observed in the distal tibia (Neumeyer et al., 2017). These ASD patients also exhibited increased body fat, increased serum IGF-1, lower lean mass, and decreased whole body and femoral neck BMD, suggesting physical activity, nutrition, and changes in IGF-1 responsiveness may contribute to ASD-related changes in bone density and microarchitecture. Increased fracture risk has also been observed in the ASD patient population. In a national study of emergency room visits, a higher rate of hip fractures was observed in both children/adolescents (3–22 years) and adults (23–50 years) with ASD compared to patients without ASD, with a higher rate of forearm and spine fractures also observed in adult women (Neumeyer et al., 2015).

While not all studies in the ASD population control for use of APTs, such as risperidone, there is evidence that patients taking APTs have decreased BMD. In a study of adolescent boys with ASD, ∼49% of patients taking an APT had hyperprolactinemia with decreased lumbar spine BMD, as well as decreased levels of the bone turnover marker, carboxyterminal cross-linking telopeptide of bone collagen, compared to patients taking APT without hyperprolactinemia (Roke et al., 2012). Another study of ASD boys aged 5–17 years taking risperidone showed decreased trabecular BMD and decreased radius bone strength compared to healthy controls (Calarge and Schlechte, 2017). Preclinical studies have shed light on the biological mechanisms by which ASD, as well as APT treatment, may impact bone health. In a genetic mouse model for human 15q11-13 duplication, decreased bone mass was observed and associated with osteoblast reduction and decreased bone formation (Lewis et al., 2017). Osteoblasts from ASD mice exhibited decreased proliferation, differentiation, and mineralization, whereas osteoclasts were minimally impacted. In a rat model of ASD, based on maternal exposure to LPS, ASD rats showed decreased bone stiffness and strength, in addition to a reduced number of OCN-positive cells compared to control rats, also indicating that ASD leads to impaired osteoblast proliferation and/or differentiation (Amini et al., 2020). Treatment of ASD rats with risperidone led to more extreme impacts on bone strength, providing evidence that APT treatment exacerbates ASD-related bone effects. Additional studies will help delineate the influence of APTs on bone health in the context of ASD.

#### Cerebral Palsy

Cerebral palsy (CP) is the most common motor disorder in children and causes a wide range of symptoms impacting neurological (e.g., ataxia, impaired gross motor coordination), orthopedic (e.g., hip dysplasia/dislocation), cognitive (e.g., autism, epilepsy), and visual/hearing systems (Brandenburg et al., 2019; Vitrikas, 2020). CP etiology is complex and involves pathophysiology in the brain and spinal cord. Spastic CP, which accounts for > 80% of cases, is thought to result from dysfunction in the spinal cord, leading to disinhibition of motor neurons and causing symptoms of spasticity, impaired coordination/movement, hyperreflexia, muscle contracture, and weakness (Sheean and McGuire, 2009; Brandenburg et al., 2019). While the pathological development of spastic CP is complex, it has been linked to asphyxia, prenatal/neonatal hemorrhagic or ischemic stroke, infection, brain malformation, trauma, and genetic factors (Brandenburg et al., 2019).

In addition to neuromuscular dysfunction, children and adolescents with CP often present with impaired bone health. Children with quadriplegic CP had reduced BMD of the lumbar spine compared to age-matched healthy children, with the most severe bone impacts observed in patients at level V of the Gross Motor Function Classification System (GMFCS) and in malnourished patients, pointing to disease severity, mobility, and malnutrition as potential risk factors for bone loss (Alvarez Zaragoza et al., 2018). In addition to disease severity, changes in the IGF-1 axis have been implicated in CP-induced bone loss. BMD was significantly decreased in children with spastic CP compared to healthy children and was associated with low circulating IGF-1 levels, severe GMFCS level, and use of anticonvulsive drugs. Children with CP also exhibited increased fracture risk (Nazif et al., 2017).

There is conflicting evidence for the role of anticonvulsive drugs as a risk factor for low BMD. In a study of non-ambulatory children with CP, no significant differences in BMD were found between children taking and not taking anticonvulsant therapies (Cheng et al., 2016). However, nutritional status was implicated as a risk factor. Studies comparing ambulatory and non-ambulatory children with CP have shown that the main predictor of low BMD in the distal femur is impaired mobility (Finbråten et al., 2015). Adolescents and young adults with CP have shown similar deficits in areal BMD, which were associated with reduced mobility by the GMFCS (Trinh et al., 2019).

Although less well-studied, there is clinical evidence that adult CP patients also exhibit bone loss. In a study of premenopausal women and men under 50 years old, BMD was significantly decreased at the lumbar spine, total hip, and femoral neck regions compared to healthy controls. For the lumbar spine and hip, BMD was associated with impaired motor ability by the GMFCS (Fowler et al., 2015). In a demographically similar population, BMD at the second metacarpal bone (mBMD) was found to be decreased and correlated with use of anticonvulsant drugs (Nakano et al., 2003). In men, abnormal calcium metabolism was also associated with lower mBMD, whereas, impaired mobility was associated with mBMD in women, suggesting the mechanisms by which CP impacts bone health may be sex-dependent and include both physical and biomolecular factors. In a study of ambulatory versus non-ambulatory adult CP patients, non-ambulatory patients had decreased BMD, lower PTH levels, and higher SOST levels compared to ambulatory patients, implicating systemic changes in hormones and bone remodeling factors in CP-related bone loss in adult patients (Shin et al., 2017). Taken together, these studies implicate disease severity, mobility status, and alterations in hormones and bone remodeling factors as critical risk factors for CP-associated bone loss.

#### Epilepsy

Epilepsy is a complex neurological disorder characterized by repeated, unprovoked seizures. Diagnosis is made upon the occurrence of two or more unprovoked seizures more than 24 h apart or one unprovoked seizure with high probability of recurrence (> 60%) over the next 10 years (Beghi et al., 2015). While the etiology of epilepsy is unknown, genetic predisposition, brain injury, and infection have been implicated as causal factors (Thijs et al., 2019). Epilepsy is a highly heterogeneous disorder, with four major types (focal, generalized, combined focal/generalized, and unknown) that can be divided based on nature of seizure onset, as well as level of awareness, motor symptoms, and non-motor symptoms (Thijs et al., 2019).

Patients with epilepsy have increased risk of developing osteoporosis and increased fracture risk that is 2–6 times higher than the general population, independent of seizure-related fractures (Diemar et al., 2019a). Decreased exercise, a more sedentary indoor lifestyle, and use of anti-epileptic drugs (AEDs), which can lead to impaired coordination and disrupted calcium/vitamin D metabolism, may contribute to poor bone health (Kobau et al., 2004; Shellhaas and Joshi, 2010; Diemar et al., 2019b). A meta-analysis of epileptic children showed a significant BMD decrease at lumbar spine, trochanter, femoral neck, and total body regions (Zhang et al., 2015). Decreased serum vitamin D and increased serum alkaline phosphatase (ALP) were also observed, suggesting abnormal vitamin D and/or calcium metabolism may contribute to decreased BMD in children with epilepsy.

Use of AEDs, especially cytochrome P450 enzyme-inducing AEDs (EIAEDs), have been associated with poor bone health in children and adult patients with epilepsy. In ambulant children with epilepsy, use of > 2 EIAEDs was shown to be a significant risk factor for low lumbar BMD (Fong et al., 2018). In a case-control study of matched-pair adolescents, epileptic patients taking AEDs had a significantly increased fracture risk and a 14% reduction in trabecular volumetric BMD compared to matched controls, demonstrating a link between AED use and poor bone health (Simm et al., 2017). A similarly designed study of same-sex twin/age-matched sibling pairs showed EIAED users exhibited a greater reduction in hip and total body BMD, which was not observed in non-enzyme-inducing AED (NEIAED) users, pointing to EIAEDs as the primary driver of bone loss in this patient population (Shiek Ahmad et al., 2017). Similarly, patients on carbamazepine (CBZ), a widely used EIAED, have increased hip and femoral neck BMD loss during initial years of therapy compared to nonusers and have increased hip BMD loss compared to users on NEIAEDs, including levetiracetam and valproate. While the mechanisms by which EIAEDs impact bone have not been fully elucidated, increased catabolism of vitamin D to inactive metabolites, decreased calcium, increased PTH, and increased bone turnover have been implicated (Pack, 2008). In a study of epilepsy patients taking CBZ for > 12 months, decreased BMD and decreased serum vitamin D were observed. A concomitant increase in OCN was observed in CBZ users, suggesting that, in addition to modulation of vitamin D, CBZ may impact bone turnover (Suljic et al., 2018). In an epilepsy rat model, CBZ was associated with decreased serum vitamin D and elevated PTH, as well as decreased BMC, impaired collagen crosslinks, and decreased microhardness, indicating CBZ therapy may affect bone strength and microarchitecture (Garip Ustaoglu et al., 2018). Although less well documented, there is evidence to suggest that some NEIAEDs, including valproate, also impact bone health in patients with epilepsy. In a meta-analysis of valproate users, BMD was found to be decreased in spine and femoral neck regions compared to healthy controls and was associated with increased serum bone-specific alkaline phosphate (BALP) (Fan et al., 2016). In addition to vitamin D deficiency, use of AEDs has been associated with hyponatremia in epilepsy patients. In a cross-sectional study of patients with epilepsy, hyponatremia was observed in ∼10% of the population and was independently associated with decreased BMD and increased risk of osteoporosis, providing evidence that altered sodium metabolism may contribute to AED-associated bone loss in epilepsy (Diemar et al., 2019a). While additional mechanistic studies are needed, current evidence points to altered vitamin D and sodium metabolism, elevated PTH, and dysregulated bone turnover in epilepsy patients taking AEDs.

### Neuromuscular Disease

#### Amyotrophic Lateral Sclerosis

Amyotrophic lateral sclerosis (ALS) is a progressive neurodegenerative disorder characterized by loss of cortical, brainstem, and spinal motor neurons that results in progressive muscle atrophy. There is currently no cure for ALS. Most patients eventually become dependent on mechanical ventilation and usually die due to respiratory failure (Portaro et al., 2018). How ALS may affect the skeleton and modify osteoporosis risk is under-studied. Altered calcium metabolism, hypovitaminosis D, reduced cortical bone mass, and vertebral defects have been noted in ALS patients (Mallette et al., 1977; Yanagihara et al., 1984; Joyce et al., 2012). In clinical studies, ALS patients had 14% more fractures than controls, and, in a Swedish population, fracture was associated with higher incidence of ALS (Parfitt, 1994; Peters et al., 2017). ALS has also been associated with increased bone turnover markers in the blood (Fang et al., 2010). In a case report of an 81-year-old man with ALS, multiple hidden vertebral fractures were found, with a low Z score but normal TBS, suggesting normal bone structure (Portaro et al., 2018). It is possible that reduced muscle strength from ALS resulted in an unsupported spinal column, leading to these fractures. In a mouse model of ALS, SOD1^*G93A*^ mice demonstrated decreased bone mass with notable whole bone biomechanical deficits (Ko et al., 2018). Osteoblasts isolated from SOD1^*G93A*^ mice with muscle atrophy had impaired differentiation capacity, while osteoclast activity was increased compared to wildtype mice (Zhu et al., 2015). Aberrant Akt, Erk1/2, SOST, RANKL, and β-catenin signaling pathways were noted, which could be further links between ALS-induced muscle atrophy and bone loss. Muscle and bone are known to cross-talk extensively, with skeletal muscle providing an important source of osteogenic growth factors (e.g., IGF-1, FGF-2), as well as driving bone morphogenesis through mechanical load (Hamrick et al., 2010; Sharir et al., 2011; Zhou et al., 2015). Thus, it is likely that muscle atrophy is the key component linking ALS to bone loss and increased fracture risk.

Another interesting mechanism that may link ALS to bone is accumulation of neurotoxic metals. Neurotoxic metals, such as lead, have been found in the brain and CSF of ALS patients and have been shown to affect bone mineralization, whereby they accumulate in the bone and act as substitutes for calcium in hydroxyapatite (Roos et al., 2013; Chen X. et al., 2014; Roos, 2014). It is also possible that osteoporosis can worsen neurodegenerative disease outcomes, as the bones and CSF share circulation. Bone may act as a sink for neurotoxic metals, releasing them during osteoporosis, thereby facilitating neurodegeneration (Roos, 2014). Patients exposed to high concentrations of neurotoxic metals should be more closely monitored for osteoporosis and frequently tested for neurotoxic metals. It is still unclear how any specific molecular mechanism tied to ALS may influence bone homeostasis or if increased risk is due primarily to lifestyle factors, as age, reduced mobility, increased falls, and weight loss are all prevalent in ALS and are common osteoporosis risk factors. Thus, more studies are needed examining molecular mechanisms linking ALS to bone, particularly any associated with muscle atrophy or neurotoxic metals.

#### Myasthenia Gravis

Myasthenia gravis (MG) is a neuromuscular disorder that results in weakening of the skeletal muscles. It is frequently a product of autoimmune disease, resulting in attack on nicotinic acetylcholine receptors or on muscle-specific tyrosine kinase (MuSK). The exact cause of this autoimmune reaction is still under investigation. Symptoms include difficulty breathing or swallowing, fatigue, drooping of eyelids, problems walking or lifting objects, trouble talking, and double vision. Regarding impacts on bone, a study found that MG resulted in a 1.96-fold increased risk of developing osteoporosis, likely due to lack of outdoor activity leading to decreased sunlight exposure/vitamin D and physical inactivity (Yeh et al., 2014). However, altered acetylcholine signaling may also play a role, as osteoblasts express acetylcholine receptors and elevated acetylcholine levels induce osteoblast proliferation (En-Nosse et al., 2009; Sato et al., 2010). In addition, MG patients prescribed antidepressants, anxiolytics, or anticonvulsants had increased fracture risk (Pouwels et al., 2013). The mechanism underlying this finding is unknown but may be due to altered neuronal signaling, as use of SSRIs reduces BMD in humans (Ducy and Karsenty, 2010; Haney et al., 2010; Brinton et al., 2019). The use of anticonvulsants increases vitamin D catabolism, leading to increased bone resorption (Kinjo et al., 2005). However, how these mechanisms coincide with MG to increase fracture risk remains unknown.

In addition to the effects of MG itself, corticosteroids, a common treatment for MG, are known to cause bone loss, with long-term corticosteroid use the most common mediator of secondary osteoporosis (Buehring et al., 2013). Corticosteroids have been shown to increase osteoporosis risk in MG (Pascuzzi et al., 1984; Konno et al., 2015; Braz et al., 2017). A case study reported an MG patient with eight spinal compression fractures due to intensive and prolonged prednisone treatment, but DEXA scanning and/or bisphosphonate treatment were not mentioned as being used prophylactically (Raibagkar et al., 2017). This situation may, unfortunately, not be uncommon among neurologists, as there may be a lack of awareness and non-implementation of iatrogenic osteoporosis treatment guidelines, with limited requested DEXA scanning and a lack of understanding in how to interpret T scores leading to over- or under-treatment with bisphosphonates (Lewis and Smith, 2001; Lozsadi et al., 2006; Gallagher and Sturrock, 2007). To demonstrate the benefits of prophylactic osteoporosis treatment, BMD in 36 MG patients who had undergone long-term prednisolone administration with concurrent treatment with elcatonin was measured, and a decrease in BMD was found in 31% of female patients and osteoporosis in 11.5% compared to a presumptive rate of 22.6% in the general population. No osteoporosis was detected in male patients (Wakata et al., 2004). This suggests that prednisolone-treated MG patients have an acceptable bone loss risk when monitored and provided prophylactic osteoporosis treatment. Likewise, MG patients with history of glucocorticoid treatment who were treated with alendronate combined with alfacalcidol showed increased BMD and decreased bone turnover biomarker levels (Lv et al., 2018). Further studies are needed to dissect the mechanistic roles by which MG impacts bone health independent of corticosteroid use.

#### Neuromuscular Dystrophy

Neuromuscular dystrophy (NMD) is a group of degenerative muscle diseases in which genetic mutations result in loss of muscle mass and progressive weakness. Muscle-bone interactions have been extensively studied, and it is thought that alterations in muscle-derived myokines, bone deformation as a result of muscle weakness, and direct effects of genetic defects on bone cells may negatively impact bone in NMD, as well as low vitamin D, nutritional deficits, immobility, and drug treatments (Kurek et al., 1996; Febbraio and Pedersen, 2002; Veilleux and Rauch, 2017).

Duchenne muscular dystrophy (DMD) is the most common form of NMD and is an X-linked recessive disorder linked to a mutation in the dystrophin gene that is characterized by progressive muscle weakness due to reduction of dystrophin and destabilizing effects on the sarcolemmal membrane, ultimately leading to premature death (Hoffman et al., 1987). There is currently no cure. DMD is the most studied form of NMD in regards to bone health, with reports dating back to 1941 (Maybarduk and Levine, 1941; Joyce et al., 2012). Boys with DMD have abnormalities in bone geometry, presenting with slender long-bone shafts, a likely risk factor for long bone fracture (Veilleux and Rauch, 2017). Up to 90% of patients with DMD have scoliosis, providing strong evidence for the role of dystrophin in regulating bone health and development (Pecak et al., 1980). Decreased BMD, increased fracture rate, and vitamin D deficiency have been reported in DMD (Siegel, 1977; Larson and Henderson, 2000; Vestergaard et al., 2001; McDonald et al., 2002; Bianchi et al., 2003; Hawker et al., 2005; Perera et al., 2016; Joseph et al., 2019). Aparicio et al. found that eight out of ten boys aged 6–11 years with DMD years had osteoporosis in the proximal femur, while the remaining two boys had osteopenia (Aparicio et al., 2002). Bianchi et al. showed that DMD patients had reductions in spine BMD, hypocalciuria, increased bone turnover markers, and low vitamin D levels (Bianchi et al., 2003). Fall risk is also increased with DMD, thereby further increasing fracture risk (McDonald et al., 2002).

Using the dystrophin-null *mdx* mouse, Rufo et al. found *mdx* mice displayed changes in BMD in a manner similar to that observed in humans. Osteoclasts and IL-6 levels were increased, while RANKL:OPG ratio was altered in favor of increased bone resorption. Human primary osteoblasts incubated with sera from DMD patients showed decreased nodule mineralization, downregulation of *OSX* and *OCN*, and upregulation of *IL6*, *IL11*, *inhibin-βA*, and *TGFβ2*, suggesting DMD can directly impact bone at a cellular and molecular level (Rufo et al., 2011). This study also proposed anti-IL-6 therapy as a possible treatment of bone disease in DMD. In regard to current DMD treatment, increasing routine use of corticosteroids to treat DMD is concerning, due to the known effects of chronic glucocorticoid treatment on bone health (Bell et al., 2017). In DMD, corticosteroid use may be increasing prevalence of vertebral fracture (King et al., 2007; Houde et al., 2008; Annexstad et al., 2019). However, robust evidence that corticosteroid use increases fracture risk in DMD, specifically, is lacking. Teriparatide has also been used with success in DMD, showing improvements in BMD and quality of life with few, if any, side effects (Catalano et al., 2016; Nasomyont et al., 2020). It is clear that routine imaging, particularly of the spine, clinical monitoring, consideration of skeletal delay, possible vitamin D supplementation and/or teriparatide treatment are needed when evaluating bone health and BMD Z-scores in patients with DMD (Ko et al., 2020).

Another form of NMD, spinal muscular atrophy (SMA), an autosomal recessive disorder due to mutations in survival motor neuron 1 and (SMN1/2) that involves selective destruction of anterior horn cells, is associated with fractures at birth and increased rates of fracture throughout life. There are multiple subtypes that present with different degrees of severity. Both long bone and vertebral fractures are relatively common, as well as scoliosis (Vai et al., 2015). Animal studies have shown that the exon 7 splice variant of SMN upregulates osteoclast-stimulating factor, promotes osteoclast formation, and enhances bone resorption (Kurihara et al., 2001; Shanmugarajan et al., 2009). However, more in depth mechanistic studies are needed in humans to determine direct effects of SMA mutations on bone.

### Psychological

#### Major Depressive Disorder

Major Depressive Disorder (MDD) is a highly prevalent, heterogeneous mood disorder characterized by biases in attending to and recalling negative emotional stimuli that align with a negative mood state (Belmaker, 2008). The etiology of MDD is complex and likely involves dysregulation of serotonin and norepinephrine neurotransmission, increased neuroinflammation, and environmental factors (Woelfer et al., 2019). Significant clinical evidence shows MDD is associated with poor bone health, including decreased BMD and increased risk for osteoporosis. Patients with MDD were found to be 1.3 times more likely to develop osteoporosis compared to patients without MDD (Lee et al., 2015). In a population-based cohort study, patients diagnosed with a depressive disorder were found to have higher risk of vertebral fracture compared to healthy patients (Lee et al., 2017). In a meta-analysis, MDD was associated with lower BMD in the lumbar spine, femur, and total hip regions in all age groups compared to healthy controls (Schweiger et al., 2016). Differences in lumbar spine and femur BMD were more prevalent in women, whereas differences in hip BMD were more prevalent in men, suggesting sex-dependent effects of MDD on bone. Clinical studies also point to age-dependent differential effects of MDD on bone. Adolescent boys with MDD had lower hip and femoral neck BMD compared to age-matched controls, whereas no differences in BMD were found in girls with MDD compared to healthy controls (Fazeli et al., 2013).

Although many clinical studies examining the impact of MDD on bone do not stratify based on MDD subtype, different subtypes of MDD may be associated with greater risk for low BMD. In a study of premenopausal woman with melancholic, atypical, or undifferentiated MDD, women with undifferentiated MDD were more likely to exhibit low BMD at the femoral neck compared to healthy control patients. Elevated levels of adrenocorticotropic hormone (ACTH) were observed in women with atypical MDD, while higher levels of leptin were observed in women with melancholic MDD, suggesting differences in the pathophysiology between MDD subtypes may also account for observed differences in bone health (Cizza et al., 2010; Woelfer et al., 2019). Psychological conditions commonly co-occurring with MDD, including anxiety, may also contribute to bone loss. In a study of postmenopausal women, patients with higher anxiety levels exhibited decreased lumbar and femoral neck BMD and increased fracture risk compared to patients with lower anxiety levels (Catalano et al., 2018). A separate study in postmenopausal women showed that anxiety level, as determined by the Hamilton Anxiety Rating Scale (HAMA), was inversely correlated with levels of vitamin D and this association was independent of patient level of depression, suggesting that anxiety independently impacts vitamin D metabolism and this may be one mechanism by which anxiety contributes to bone loss and decreased patient quality of life in the aging population (Martino et al., 2018a,b). As SSRIs are used as a first-line therapy for MDD patients and have negative bone effects, it is difficult to parse out how MDD pathophysiology, versus SSRI treatment, impacts bone health. Recurrent MDD in adult men was associated with decreased forearm and total body BMD compared to men with no history of MDD (Rauma et al., 2015). Further, antidepressant use in this population was associated with lower BMD in lower-weight men only. Interestingly, acute MDD episodes were found to be associated with higher BMD at total hip, pointing to differential effects of acute versus recurrent MDD. In a study of adolescents and young adults who were either unmedicated or within 1 month of starting SSRI treatment, SSRI use was associated with increased lumbar spine areal BMD in females and decreased lumbar spine areal BMD in males, suggesting SSRIs independently impact bone health in a sex-dependent manner in MDD (Calarge et al., 2014). In young to middle-aged patients with an acute episode of depression, SSRI use had no impact on BMD (Malik et al., 2013). However, increased levels of OPG and increased levels of physical activity were observed in MDD patients, suggesting there may be protective/compensatory mechanisms in acute MDD that temper SSRI-mediated bone effects. In a study examining levels of bone turnover markers in medication-free inpatients with recurrent MDD, patients with MDD exhibited decreased levels of baseline OPG/RANKL and plasma OPN, indicating MDD may modulate bone physiology independent of SSRI use (Kadriu et al., 2018).

Bone loss in MDD patients may also be associated with increased activation of the ACTH and PTH axis, dysregulation of the OPG-RANK-RANKL axis, increased inflammation, and autonomic dysfunction (Rosenblat et al., 2016; Elefteriou, 2018). In postmenopausal women, patients with MDD exhibited decreased BMD and increased levels of PTH and RANKL compared to healthy controls (Atteritano et al., 2013). In premenopausal women, patients with MDD exhibited lower BMD at baseline, as well as increased PTH and ACTH at baseline and 6-month follow-up compared to healthy controls (Cizza et al., 2012). Levels of PTH remained higher in MDD patients at 24 months, suggesting sustained activation of the PTH axis may perpetuate negative bone effects. Calcium and vitamin D levels were also significantly decreased in patients with MDD compared to controls, indicating vitamin deficiency is a contributing factor. Increased prevalence of low BMD at the femoral neck and hip was observed in premenopausal women with MDD compared to controls, with a concomitant increase in circulating proinflammatory cytokines (e.g., IL-1β, IL-2, IL-6, TNF-α) (Eskandari, 2007). Significant increases in plasma IL-6 and changes in its diurnal release were also observed in patients with active MDD and found to correlate with mood ratings, providing additional evidence that inflammation may dually mediate neurocognitive effects and bone effects in MDD (Alesci et al., 2005). Patients with MDD also commonly present with autonomic dysfunction (Kemp et al., 2010; Yang et al., 2011; Brunoni et al., 2013). Studies in preclinical models show that MDD-associated trabecular bone loss is associated with increased levels of bone norepinephrine and can be attenuated by treatment with propranolol, a beta-adrenergic antagonist (Yirmiya et al., 2006). Together, these studies implicate increased sympathetic output as a driver of MDD-associated bone loss, although additional clinical studies will be necessary to determine the mechanisms by which altered sympathetic signaling impacts bone in MDD patients. Further, additional clinical studies that carefully control for concomitant SSRI use, MDD subtype and nature (acute versus recurrent), and comorbid conditions such as anxiety, will better our understanding of MDD-driven bone pathophysiology.

#### Post-traumatic Stress Disorder

Post-traumatic stress disorder (PTSD) is characterized by an abnormal, persistent response to a traumatic event (Seal, 2007; Heron-Delaney et al., 2013). Patients with PTSD exhibit re-experiencing symptoms related to the initial traumatic event, such as flashbacks and nightmares, as well as avoidance, arousal/reactivity, cognitive, and mood symptoms, which can manifest as being easily startled, difficulty sleeping, negative thoughts, and angry outbursts (Qi et al., 2016). While the pathophysiology of PTSD is complex and not fully understood, dysregulations in the HPA axis and glucocorticoid signaling, as well as increased neural and systemic inflammation, have been implicated in disease initiation and development (Girgenti et al., 2017; Dunlop and Wong, 2019).

In addition to primary psychological symptoms, studies in adult, elderly adult, Veteran, and Prisoner of War populations provide substantial evidence that PTSD negatively impacts bone health (Glaesmer et al., 2011; Hain et al., 2011; Huang et al., 2018). A large nationwide longitudinal study showed adults with PTSD were more likely to develop osteoporosis at an earlier age, compared to age-matched, healthy controls (Huang et al., 2018). Similarly, the National Health and Resilience in Veterans study showed that U.S. Veterans diagnosed with PTSD had significantly increased risk for developing osteoporosis or osteopenia (El-Gabalawy et al., 2018). Evidence also supports that PTSD patients have an increased fracture risk (Jiang et al., 2018).

While the mechanisms by which PTSD impacts bone health have not been fully elucidated, several molecular, hormonal, and immune-related pathways implicated in PTSD pathophysiology negatively impact bone and have been extensively reviewed elsewhere (Kelly et al., 2019). In response to a stressor, stimulation of the glucocorticoid axis can lead to activation of inflammatory and RANK pathways via NF-kB, decreased osteoblast function, and prolonged osteoclast viability (Vega et al., 2007; Briot and Roux, 2015). Stress-induced increases in catecholamines, the “fight-or-flight” hormones, can also lead to activation of β-adrenergic receptors, stimulating RANKL expression and promoting osteoclast differentiation (Rodrigues et al., 2012). In a mouse model of PTSD, PTSD was associated with significantly decreased BMC and BMD in the femur, lumbar vertebra, and tibia 3 weeks following initial trauma, providing evidence that activation of the stress response in PTSD mice leads to bone loss (Yu et al., 2012). In addition to dysregulated glucocorticoid and stress hormone signaling, changes in IGF signaling and immune activation may contribute to PTSD-related bone loss. IGFs have been implicated in stress signaling and are known to be important regulators of the osteoblast-osteoclast balance (Canalis, 2009; Zegarra-Valdivia, 2017). IGF-1 supports osteoblast differentiation, with decreasing levels of IGF-1 in the aging population being associated with osteoporosis (Perrini et al., 2010; Crane et al., 2013). Increased levels of inflammatory factors, including IL-1β, IL-6, and TNF-α, are upregulated in patients with PTSD and have been implicated in mediating neurocognitive effects of the disorder (Passos et al., 2015; Lindqvist et al., 2017; Imai et al., 2018). Proinflammatory cytokines also promote osteoclast formation and amplify bone resorption (Sims, 2016; Weitzmann, 2017). These studies suggest PTSD-induced inflammation may negatively impact bone health, although additional studies are needed to delineate these effects.

In addition to shared mechanisms of pathophysiology, therapies used to treat PTSD may also negatively impact bone health. First- and second-line treatment options for PTSD patients, including SSRIs and antipsychotic drugs, are associated with increased risk of developing osteoporosis and fracture (Vestergaard et al., 2006, 2008; Haney et al., 2010; Alexander, 2012; Rabenda et al., 2013; Seifert and Wiltrout, 2013; Rauma et al., 2015). Therefore, it is reasonable to speculate that SSRI use may exacerbate PTSD-associated bone loss. However, clinical evidence of the combined effects of PTSD and SSRI use on bone health is currently lacking.

#### Substance Abuse and Addiction

Substance use disorders (SUDs), including addictions to alcohol, heroin, cannabis, ecstasy, cocaine, and amphetamines, are a major global health concern. While addiction to different substances can produce different neurobiological effects, there are shared layers of symptomology between different substance groups. These include a reduction in response to normal biological activities like social cooperation and a loss of ability to control drug-seeking behavior. Progressive changes in dopamine transmission in the corticolimbic brain regions, alterations in glutamatergic synapses, and environmental stress have been implicated in the initiation and development of SUDs (Reid et al., 2012).

In addition to changes in neural circuitry and plasticity, SUDs have been associated with impaired bone health. The impact of alcohol addiction on bone has been well studied and will be the focus of this section, although opioid, cannabis, and amphetamine addictions have also been associated with decreased BMD and increased fracture risk (Gozashti et al., 2011; Mosti et al., 2016; Gotthardt et al., 2017; Heydari et al., 2017; Sophocleous et al., 2017). Chronic-excessive use of alcohol has been shown to negatively impact bone health, resulting in decreased BMD, decreased BMC, and increased fracture risk. In a study of males classified as heavy drinkers, osteopenia was observed in 23% of the population, with a significant inverse correlation observed between total alcohol intake and BMD (González-Calvin et al., 2009). In another study of chronic alcoholic males, 34% of patients had osteoporosis, with low BMD in the femoral neck and lumbar spine compared to age-matched, healthy controls (Peris et al., 1995). In addition to increased risk of vertebral and non-vertebral fractures, chronic male alcoholics exhibited increased ALP and BALP, as well as significantly reduced vitamin D and BGP levels (Santori et al., 2008). Together, these studies point to impaired bone formation as the major mechanism by which alcohol addiction impacts bone. Although less well studied, chronic female alcohol users also exhibit impaired bone health. In a cross-sectional study of women aged 18–70 years, chronic alcohol users exhibited decreased femoral neck and lumbar spine BMD and had a higher prevalence of fractures compared to non-alcohol-abusing women, although fracture risk could not be attributed to alcohol use alone (Clark et al., 2003). In a study of both male and female chronic alcohol users, BMD directly correlated with total cholesterol and LDL-cholesterol, independent of liver function, providing evidence that alcohol addiction may mediate changes in lipid profiles that have separate effects on bone health. Together, these studies provide evidence that alcohol abuse negatively impacts bone health and is closely intertwined with sex, overall nutrition, and vitamin D deficiency.

Due to the nature of SUDs, it is difficult to parse out the effects of substance versus substance-induced neurological changes on bone. *In vitro* studies provide evidence that alcohol can directly impact the osteoblast:osteoclast balance. Treatment of BM cultures with alcohol or acetaldehyde directly impaired osteoblast differentiation, decreased osteogenesis, and promoted adipogenesis (Giuliani et al., 1999; Cui et al., 2011). Further, ethanol treatment increased bone resorption by osteoclasts *in vitro* (Cheung et al., 1995). In a rabbit model of alcoholism, increased triglyceride-bearing osteocytes and increased empty lacunae were observed, suggesting alcohol leads to impaired osteocyte function and may promote osteocyte apoptosis (Wang et al., 2003). Preclinical studies also point to a role for alcohol-induced changes in leptin signaling, resulting in impaired osteogenesis and increased adipogenesis (Wang et al., 2003; Otaka et al., 2007; Maurel et al., 2011). In alcoholism and other SUDs, it is likely that both direct effects of the substance itself and indirect effects (e.g., neurobiological impacts on bone turnover) are responsible for impaired bone health and will require further study to fully dissect.

### Trauma and Spinal Cord

#### Acute Spinal Cord Injury

The global incidence of spinal cord injury (SCI) is 10.4–83 cases/million/year. Bone loss below the level of the lesion is rapid and as high as 4% per month in trabecular bone and 2% per month in cortical bone (Wilmet et al., 1995; Szollar et al., 1997; Dauty et al., 2000). This bone loss persists for ∼2 years post-SCI, with peak loss at 3–5 months, resulting in increased risk of fracture and osteoporosis (Roberts et al., 1998; Maïmoun et al., 2006; Smith and Carroll, 2011). Post-SCI, bone resorption markers increase with a lack of concomitant increase in bone formation markers (Smith and Carroll, 2011; Thakkar et al., 2020). Numerous mechanisms have been implicated in SCI-related bone loss, including mechanical unloading from loss of motor function, as well as metabolic, endocrine, neural denervation, and vascular changes (Jiang et al., 2006b). Each of these can result in osteoblast:osteoclast imbalance, leading to bone loss, osteoporosis, and fragility fractures. In the absence of mechanical strain, osteocytes signal to reduce osteoblast activity, resulting in reduced bone formation (Jiang et al., 2006a,b). SCI also impacts the OPG-RANKL system and Wnt signaling, shifting the balance to bone resorption over bone formation (Maïmoun et al., 2006; Bonewald and Johnson, 2008). Altered vasoregulation due to injury impacts viability of oxygen and nutrients to bone, promoting osteoclast formation and bone resorption (Jiang et al., 2006a,b). Decreased innervation due to SCI may also affect availability of neuropeptides, such as vasoactive intestinal peptide (VIP) and calcitonin gene-related peptide (CGRP), which suppress bone resorptive activities through RANKL/OPG pathway (Yoon et al., 2004). Decreased PTH has also been reported 4–12 months post-SCI, which can lead to decreased vitamin D and subsequent impaired absorption of dietary calcium (Giangregorio and Blimkie, 2002; Jiang et al., 2006a,b). Thus, while disuse is considered to be the most impactful factor in post-SCI osteoporosis and associated fractures, it is clearly a more complex, multi-factorial process, and this should be considered when developing therapeutic strategies.

#### Traumatic Brain Injury

Traumatic brain injury (TBI) results in temporary to permanent neurological damage and dysfunction and is associated with increased mortality and morbidity. TBI is often referred to as the “silent epidemic,” and, while the incidence has been difficult to determine, reports suggest 69 million individuals suffer TBI from all causes annually (Dewan et al., 2019). Patients with TBI exhibit an elevated risk for fracture and reduced BMD. A recent study demonstrated that patients recovering from TBI had suboptimal BMD measurements that were low for their age and gender, with 18% of the participants meeting criteria for osteopenia measured at the radius and 51% meeting the criteria for osteopenia/osteoporosis measured at the tibia (Banham-Hall et al., 2013). Markers of bone turnover, including OCN, type I collagen, and PTH, were dysregulated during the early post-traumatic period, suggesting an imbalance between bone formation and resorption that occurred rapidly post-injury (Trentz et al., 2005). Recent preclinical studies suggest inflammatory stress on bone and BM following TBI leads to NFκB activation, which, in turn, induces osteoclastogenesis and bone resorption (Singleton et al., 2019). Pre-clinical studies from Mohan’s group using a repetitive mouse TBI model have shown reduced BMC, bone area, bone strength, and BMD in TBI mice accompanied by negative impacts on cortical structure and trabecular architecture (Yu et al., 2014a,b). Their work also demonstrated that mild TBI and bony effects were associated with decreased circulating IGF-1 levels (Yu et al., 2014a). Pituitary dysfunctions post-TBI are common, present in 25–70% of patients (Rosario et al., 2013). Deficiencies in pituitary hormones, including those that impact bone formation and contribute to peak bone mass (e.g., IGF-1), can result in negative effects on skeletal maintenance (Mohan et al., 2003; Mohan and Baylink, 2005; Xing et al., 2012). An additional contributor to low BMD risk in TBI patients is the use of anti-epileptic drugs, which induce the cytochrome P450 system and likely increase the conversion of vitamin D to its inactive forms, resulting in less biologically active vitamin D, decreased calcium absorption, hypocalcemia, increased PTH levels, and compensatory mobilization of calcium stores from bone (Smith et al., 2016). Polytrauma with TBI and concomitant fracture has been shown to result in higher functional deficits and mortality rates (Albrecht et al., 2019). Studies using two mouse models of TBI showed that neurological inflammation and brain damage was increased in animals with fracture and that this damage could be alleviated by blocking the inflammatory effects of fracture (Shultz et al., 2015; Yang et al., 2016). While mechanisms driving the effects of fracture on TBI outcomes are still being elucidated, these studies suggest that exacerbated neuroinflammation may be an important contributing factor.

### Vascular Disease

#### Stroke

Stroke is one of the most common neurologic problems, with hemiplegia being a common outcome that results in loss of voluntary movement, immobilization, and sensory disturbances. There is a robust and long literature examining the effects of stroke on bone health and fracture, notably in the hip (Peszczynski, 1957). The relative risk of fracture after hospitalization for stroke is greater than 7 times the rate of fracture in age- and sex-matched control populations (Kanis et al., 2001). BMD is lower following stroke, while low BMD may also be a prospective risk factor for stroke (Ramnemark et al., 1999; Jørgensen et al., 2000; Myint et al., 2007, 2014; Lee et al., 2013; Lam et al., 2016). Low BMD may increase stroke risk through altered estrogen/OPG signaling that leads to increased risk of intracerebral hemorrhage (Strand et al., 2007). Cibelli et al. showed that aseptic long bone fracture caused neuroinflammation and cognitive decline (Cibelli et al., 2010). Thus, determining the cause-and-effect relationship between stroke and fracture can be complex. A meta-analysis showed that, in subacute and chronic stroke, skeletal sites in the affected/paretic limbs had greater decline in bone quality and deleterious changes in bone geometry compared to unaffected/non-paretic limbs. This rate of change slowed as post-stroke duration increased, with the greatest changes occurring in the first few months post-stroke. A strong relationship between bone density/strength index and muscle strength/mass was also noted, demonstrating the importance of muscle-bone interactions and how they may act as a functional unit, as proposed by Schoenau (2005). These findings suggest muscle strength training and early intervention are key to minimizing negative bone effects of stroke (Borschmann et al., 2018; Yang et al., 2020). To this end, a mix of resistance, aerobic, and dynamic loading exercises resulted in better bone outcomes in the hip and tibia on the affected side in chronic stroke patients (Pang et al., 2005, 2006).

Muscle imbalance may not be the only mechanism by which stroke affects bone health. Lower bone turnover markers have been noted in serum from stroke patients, suggesting dysregulated remodeling at the bone multicellular unit (Sato et al., 2000). Stroke patients also have higher energy expenditure, with this interruption of energy homeostasis potentially negatively impacting the skeleton during bone remodeling (Detrembleur et al., 2003; Lee et al., 2007; Driessler and Baldock, 2010). Additionally, reduced vitamin D levels, degree of recovery, increased fall risk, and use of anticoagulants may increase bone loss post-stroke (Jørgensen et al., 2000; Smith and Carroll, 2011; Batchelor et al., 2012; Signorelli et al., 2019). Thus, increased bone screening measures are needed in stroke patients, as screening may currently be infrequent (Kapoor et al., 2019). Osteoporosis treatments, such as bisphosphonates, may be beneficial for preserving BMD post-stroke, but there is little evidence to date (Hsieh et al., 2020). More studies are needed to dissect the molecular mechanisms at the intersection of bone and stroke to guide treatment and screening recommendations.

### Other Disorders

#### Chronic Fatigue

Chronic fatigue syndrome (CFS) is a complex neurological disorder associated with persistent, overwhelming fatigue that affects > 3% of the population in Western countries and is more prevalent in women (Griffith and Zarrouf, 2008). Diagnostic criteria include severe, persistent fatigue for at least 6 months, exclusion of other medical disorders, and observation of at least four minor symptoms, including impaired memory, nausea, extreme post-exertion fatigue, headaches, muscle pain, sore throat, and poor sleep (Committee on the Diagnostic Criteria for Myalgic Encephalomyelitis/Chronic Fatigue Syndrome et al., 2015). There remains a lack of treatment and diagnostics tools for CFS, although glucocorticoids have been used (McKenzie et al., 2000). Bone loss and increased fracture risk have been reported in individuals with CFS, independent of glucocorticoid use. Hoskin et al. found that hip BMD was approximately 7% lower in women with CFS (Hoskin et al., 2006). A prospective study reported a 1.16-fold increased risk of fracture in the CFS cohort without osteoporosis compared to the non-CFS cohort (Chen C.-S. et al., 2014). However, no mechanistic insights were provided in these studies. Other studies have reported that IGF-1 levels are altered in CFS patients (Buchwald et al., 1996; Berwaerts et al., 1998; Cleare et al., 2000; Nijs et al., 2003). IGF-1 is essential for osteoblast proliferation, thus impaired secretion could lead to bone loss. Studies are needed to further characterize those CFS patients with low serum IGF-1 to determine if these subgroups have increased fracture risk compared to CFS patients with normal or high levels of IGF-1. High prevalence of mycoplasma infections has also been reported in CFS patients (Choppa et al., 1998; Nasralla et al., 1999), which can stimulate macrophage activation and release of pro-inflammatory cytokines that enhance osteoclast activity. *M. fermentans* has been shown to produce 2-kDa macrophage-activating lipopeptide (MALP-2), which stimulates macrophages and bone resorption in a dose-dependent manner and is increased with CFS (Piec et al., 1999). Thus, chronic fatigue may induce bone loss or increase fracture risk through increased inflammation and/or dysregulation of growth factors.

#### Sleep Disorders

Chronic sleep deprivation is becoming a widespread problem, with at least one-third of adults reporting less than 6.5 h of sleep per night compared to about 9 h of average sleep in the early 1900s (Bonnet and Arand, 1995; Spiegel et al., 1999; Specker et al., 2007). Sleep deprivation can negatively impact health by decreasing cardiovascular health and increasing risk for development of diabetes and obesity. Since circadian rhythm has been extensively shown to regulate bone, sleep disorders may also affect bone homeostasis (Swanson et al., 2015, 2018; Song et al., 2018). Spiegel et al. found that cortisol concentrations were higher in the evening with sleep deprivation, a known risk factor for bone loss (Spiegel et al., 1999). Three weeks of sleep restriction has been shown to cause a decline in N-terminal propeptide of type 1 procollagen (P1NP), a bone formation marker, with stable resorption markers (e.g., CTX), suggesting an uncoupling of bone remodeling. Importantly, this observed decrease in P1NP was not rescued with ongoing exposure, suggesting BMD could be lowered over time when paired with poor sleeping habits (Swanson et al., 2017, 2019). Sleep-deprived women had lower cortical BMD compared to women with normal sleeping habits, and sleep quality, sleep latency, and sleep timing, but not sleep duration, were associated with osteopenia and sarcopenia in middle-aged individuals (Specker et al., 2007; Lucassen et al., 2017). Shiftwork may also lower BMD, providing evidence that circadian rhythm, and not just amount of sleep, can significantly impact bone (Quevedo and Zuniga, 2010; Kim et al., 2013).

Obstructive sleep apnea (OSAS) has also been shown to lower BMD and vitamin D levels, possibly due to increased hypoxia, which can cause oxidative stress, SNS activity, endothelial dysfunction, and stimulation of osteoclasts (Shahar et al., 2001; Arnett, 2010; Uzkeser et al., 2013; Terzi and Yılmaz, 2016; Eimar et al., 2017). Further, OSAS increases systemic inflammation, with resultant increased IL-6, TNF-α, and C-reactive protein (CRP) production, known risk factors for bone loss (Tomiyama et al., 2008). Fracture can also affect sleep disturbance, likely due to effects on emotional well-being (Shulman et al., 2015). Likewise, vertebral fractures have been associated with poor sleep, and those with osteoporosis were 67% more likely to report decreased sleep. These studies suggest a negative feedback loop may be occurring between reduced sleep and poor bone health (Silverman, 1992; Foley et al., 2004). Interestingly, Cikrikcioglu et al. found that women with restless legs syndrome had increased lumbar BMD, despite lower vitamin D levels, possibly due to unconsciously performing exercise (Cikrikcioglu et al., 2016).

Thus, there are many mechanisms that may link disrupted sleep to bone loss, including increased systemic inflammation, hypoxia, insulin resistance, and oxidative stress, as well as altered circadian rhythm, decreased growth hormone secretion, and physical inactivity. Large-scale, prospective studies are needed to elucidate if sleep loss and/or OSAS are independent risk factors for osteoporosis (Schiza et al., 2013). Further, other sleep disorders, such as narcolepsy or somnambulism, need to be studied in more detail for their potential effects on bone.

#### Vertigo

Vertigo is a symptom in which someone feels like they are moving or surrounded by moving objects when they are not. This can be associated with nausea, sweating, vomiting, hearing loss, and/or difficulties in walking and balance. As bone mediates hearing and movement and vestibular changes alter SNS output, vertigo may be linked to alterations in bone (Radaei and Gharibzadeh, 2013; Mendy et al., 2014; Yates et al., 2014). Although there are many types of vertigo, one of the most common types, benign paroxysmal positional vertigo (BPPV), has been studied in the context of BMD and vitamin D changes. BPPV is a vestibular dysfunction that is typically unilateral and characterized by short, intense episodes of vertigo. BPPV represents 20–30% of dizziness diagnoses, with no current consensus on its etiology and pathogenesis (Grill et al., 2014; Bazoni et al., 2020). However, the incidence of BPPV increases with age and is believed to involve abnormal stimulation of the cupula by otoliths in any of the three semicircular canals upon changes in head position (Furman and Cass, 1999).

Some studies have noted that reductions in bone mass correlate with both occurrence and recurrence of BPPV (Vibert et al., 2003; Jang and Kang, 2009; Jeong et al., 2009; Kim et al., 2017; Wu et al., 2018; Wang et al., 2020). A meta-analysis found significantly higher incidence of osteoporosis and osteopenia in BPPV patients (He et al., 2019). In addition, BPPV has been associated with vitamin D deficiency, which can affect both bone and the inner ear (Jeong et al., 2013). BPPV frequently occurs in females over 50 years old, suggesting estrogen loss may be involved in onset (Vibert et al., 2003). Incidence of BPPV recurrence was significantly higher in post-menopausal women with osteoporosis (56.3%) than those with normal BMD (16.1%), and frequency of recurrence increased with decreasing BMD (Yamanaka et al., 2013). Estrogen deficiency can cause low bone mass by altering calcium metabolism, inducing a calcium insufficiency (Riggs et al., 1998). Calcium is important in the synthesis and absorption of otoconia and otoliths, which mature by absorbing calcium. Thus, if there is a shortage of calcium with reduced bone mass, incomplete maturation of otoliths could occur, leading to BPPV. The otolith also acts as a calcium reservoir to maintain calcium homeostasis when necessary, such as in postmenopausal women with osteoporosis (Campos et al., 1999). Thus, altered calcium metabolism caused by decreased estrogen secretion may be a pathophysiological mechanism shared by both BPPV and osteoporosis. Additionally, as electrical signals from the inner ear are relayed to the CNS to maintain body balance and vestibular dysfunction alters SNS output, vertigo may affect bone remodeling and bone mass, as well as fall risk, leading to increased fracture risk. In support of this, fracture risk has been shown to be increased with BPPV (Nakada et al., 2018). Likewise, the reduction in bone mass caused by vertigo may further alter calcium metabolism, increasing vertigo incidence and creating a negative feedback loop. Osteoporosis is, therefore, a risk factor for BPPV recurrence and prognosis may be clinically predicted by BMD reduction, while BPPV itself may increase osteoporosis-related fracture incidence.

## Conclusion

**FIGURE 1:**
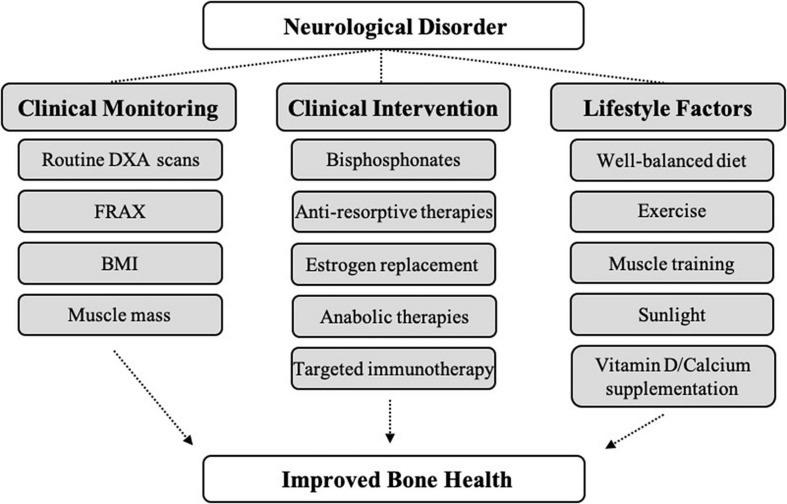
Proposed methods to improve bone health in patients with neurological disorders. There is considerable clinical evidence demonstrating negative impacts of neurological diseases on bone across many disease categories. Patients with neurological diseases exhibit decreased BMD, as well as increased risk for osteoporosis and fracture. Careful clinical monitoring, clinical intervention, and positive lifestyle changes may lead to better bone outcomes in certain subsets of neurological disease patients.

It is becomingly increasingly clear that bone is a dynamic organ with complex signaling responses throughout the body. Recent studies have led to a better understanding of the brain-bone axis, which regulates skeletal metabolism, hormonal response, and sensory innervation. In this review, we discussed how different neurological disorders impact bone health and how bone itself can affect cognitive function and development. Across many subcategories of neurological disease, there is direct pre-clinical and clinical evidence that deficits in the brain can cause deficits in the bone, including osteopenia/osteoporosis and increased fracture risk. This is due to a complex mixture of neuronal (e.g., SNS/PNS dysregulation), psychological (e.g., HPA/stress), mechanical (e.g., muscle-bone interactions), cellular (e.g., macrophage, neuron, osteoblast, osteoclast), molecular (e.g., IGF-1, IL-6, PTH, Wnt), lifestyle (e.g., falls, malnutrition, physical inactivity, vitamin D deficiency), and treatment (e.g., AED, glucocorticoid, SSRI) factors. Current treatments for osteoporosis, including bisphosphonates, estrogen replacement therapy, and anabolic therapies (e.g., teriparatide, romosozumab) may be beneficial for certain subsets of patients with neurological diseases (Figure 1). A better understanding of the mechanisms that lead to bone loss in neurological disorders is of clinical importance and may better inform treatment approaches, encourage lifestyle change, and aid in development of novel osteoporosis therapies. Similarly, a better understanding of how bone regulates the brain will provide new insights into the etiology and development of neurological disorders. Clinicians should consider taking a whole-body approach when treating neurological patients and ensure that treatments directed at the brain (e.g., glucocorticoids) are not causing deleterious effects elsewhere in the body, such as in the skeleton, which may concurrently signal through negative feedback loops to impact disease severity.

## Author Contributions

RK and SS: conception and design, drafting, and revising of the manuscript. AL: drafting and revising of the manuscript. All authors read and approved the final manuscript.

## Conflict of Interest

The authors declare that the research was conducted in the absence of any commercial or financial relationships that could be construed as a potential conflict of interest.

## Nomenclature

Aβ: Amyloid-β
ACTH: Adrenocorticotropic Hormone
AD: Alzheimer’s Disease
AED: Anti-Epileptic Drug
AKT: Protein Kinase B
ALP: Alkaline Phosphatase
ALS: Amyotrophic Lateral Sclerosis
ApoE: Apolipoprotein E
App: Amyloid Precursor Protein
APT: Antipsychotic Therapy
ASD: Autism Spectrum Disorder
BALP: Bone-Specific Alkaline Phosphatase
BBB: Blood-Brain Barrier
BGP: Bone GLA Protein
BM: Bone Marrow
BMC: Bone Mineral Content
BMD: Bone Mineral Density
CBZ: Carbamazepine
CFS: Chronic Fatigue Syndrome
CGRP: Calcitonin Gene-Related Peptide
CNS: Central Nervous System
CP: Cerebral Palsy
CRP: C-Reactive Protein
CSF: Cerebrospinal Fluid
CTX: C-Terminal Telopeptide Of Type I Collagen
DEXA: Dual-Energy X-Ray Absorptiometry
DMD: Duchenne Muscular Dystrophy
DRN: Dorsal Raphe Nucleus
EBV: Epstein-Barr Virus
EDSS: Expanded Disability Status Scale
EIAED: Enzyme-Inducing Anti-Epileptic Drug
ERK: Extracellular Signal-Regulated Kinase
FDRA: Friedreich Ataxia
FRAX: Fracture Risk Assessment
GMFCS: Gross Motor Function Classification System
GPR158: G Protein-Coupled Receptor 158
HAMA: Hamilton Anxiety Rating Scale
HPA: Hypothalamic-Pituitary-Adrenal
IκB-α: Nuclear Factor Of Kappa Light Polypeptide Gene Enhancer In B-Cells Inhibitor, Alpha
IGF-1: Insulin-Like Growth Factor-1
IL: Interleukin
LDL: Low-Density Lipoprotein
LRP5/6: Lipoprotein-Receptor-Related Protein-5/6
MALP-2: 2-kDa Macrophage-Activating Protein
mBMD: Metacarpal Bone Mineral Density
MCI: Mild Cognitive Impairment
MDD: Major Depressive Disorder
MG: Myasthenia Gravis
MJD: Machado-Joseph Disease
MS: Multiple Sclerosis
MuSK: Muscle-Specific Tyrosine Kinase
NEIAED: Non-Enzyme-Inducing Antiepileptic Drug
NF-κB: Nuclear Factor Kappa B
NFT: Neurofibrillary Tangle
NMD: Neuromuscular Dystrophy
OCN: Osteocalcin
OPG: Osteoprotegerin
OPN: Osteopontin
OSAS: Obstructive Sleep Apnea
OSX: Osterix
P1NP: N-Terminal Propeptide Of Type I Collagen
PD: Parkinson’s Disease
PNS: Parasympathetic Nervous System
PTH: Parathyroid Hormone
PTSD: Post-Traumatic Stress Disorder
RANK: Receptor Activator of Nuclear Factor Kappa B
RANKL: Receptor Activator of Nuclear Factor Kappa B Ligand
SAD: Substance Use Disorder
SAE: Sepsis-Associated Encephalopathy
SCI: Spinal Cord Injury
SMA: Spinal Muscular Atrophy
SMN: Survival Motor Neuron
SNS: Sympathetic Nervous System
SOST: Sclerostin
SSRI: Selective Serotonin Reuptake Inhibitor
SUD: Substance Use Disorder
TBI: Traumatic Brain Injury
TBS: Trabecular Bone Score
TGF-β2: Transforming Growth Factor Beta 2
TNF-α: Tumor Necrosis Factor Alpha
TREM2: Triggering Receptor Expressed On Myeloid Cells-2
VIP: Vasoactive Intestinal Peptide
Wnt: Wingless-Type Murine-Mammary-Tumor Virus Integration Site.
